# Targeting the atypical chemokine receptor 2 (*Ackr2*) improves the benefit of anti-PD-1 immunotherapy in melanoma mouse model

**DOI:** 10.1080/2162402X.2025.2494426

**Published:** 2025-04-18

**Authors:** Muhammad Zaeem Noman, Martyna Szpakowska, Malina Xiao, Ruize Gao, Kris Van Moer, Akinchan Kumar, Markus Ollert, Guy Berchem, Andy Chevigné, Bassam Janji

**Affiliations:** a Tumor Immunotherapy and Microenvironment (TIME), Department of Cancer Research, Luxembourg Institute of Health (LIH), Luxembourg City, Luxembourg; b Immuno-Pharmacology and Interactomics, Department of Infection and Immunity, Luxembourg Institute of Health (LIH), Esch-sur-Alzette, Luxembourg; c Department of Hemato-Oncology, Centre Hospitalier du Luxembourg, Luxembourg City, Luxembourg; d Department of Life Sciences and Medicine (DLSM), University of Luxembourg, Esch-sur-Alzette, Luxembourg

**Keywords:** Cancer immunotherapy, ACKR2, immune checkpoint blockade, anti-PD-1, melanoma, combination immunotherapy, immune cell infiltration, inflammatory chemokines, tumor microenvironment, CCL5, scavenger receptor, D6

## Abstract

Immune checkpoint blockade (ICB) therapies, such as anti-PD-1, have transformed cancer treatment, but many patients do not respond due to a non-inflammatory tumor microenvironment (TME). Here, we investigated the impact of targeting Atypical Chemokine Receptor 2 (*ACKR2*), which scavenges key chemokines involved in immune cell recruitment, on the improvement of anti-PD-1-based therapy. In a melanoma mouse model, we demonstrated that *Ackr2* inhibition increases the release of proinflammatory chemokines CCL5 and CXCL10 and enhances the infiltration of NK cells, activated CD8+ and CD4+ effector T cells while reducing regulatory T cells (Tregs) in the TME. Targeting *Ackr2* led to tumor growth inhibition, improved survival, and enhanced response to anti-PD-1 therapy. In BRAF- and NRAS-mutant melanoma patients, low *ACKR2* expression or high CCL5/CXCL10 levels correlated with improved survival and higher CD8+ T cell markers. Targeting *ACKR2* represents a promising approach for developing combination therapies, particularly for ‘cold’ ICB resistant tumors.

## Introduction

Cancer immunotherapies based on immune checkpoint blockades (ICBs), including antibodies blocking programmed death 1 (PD-1) or programmed death ligand 1 (PD-L1), are groundbreaking treatments for several advanced and highly aggressive cancers, such as melanoma, where conventional therapies have failed. However, clinical data show that only a small percentage of patients experience significant remissions with ICBs, while most achieve only short-term benefits or no response at all. To improve ICB efficacy, therapeutic strategies are increasingly focusing on combining multiple ICBs. The main drawback of this approach is the cytotoxic side effects resulting from disruption of the fine-tuned balance of the immune system.^1^ Therefore, novel combination strategies incorporating small molecules to enhance ICB efficacy in non-responders hold great promise for expanding their use across a broader population of cancer patients and improving survival outcomes.

One of the key factors responsible for the lack of responsiveness to anti-PD-1/PD-L1 therapies is the limited infiltration of cytotoxic immune cells into the tumor microenvironment (TME).^2^ Such infiltration strongly relies on the establishment of an inflamed TME.^3^ Based on their inflammatory status, solid tumors are classified as “hot,” displaying a T cell-inflamed signature indicative of a preexisting adaptive immune response, or “cold,” characterized by a lack of T cell infiltration and absence of such a response.^4^ Therefore, therapeutic strategies aimed at converting “cold” tumors into “hot” ones have inspired significant interest to enhance the efficacy of ICB therapies and extend their use to large number of patients and tumor types.

In melanoma, the presence of lymphocytes has been demonstrated to be closely associated with the expression of a well-defined chemokine signature, including CCL2, CCL4, CCL5, CXCL9, CCL19, CCL21, CXCL10, CXCL11, CXCL13, and XCL2. Conversely, deficiencies in these chemokines within the melanoma TME was shown to limit the recruitment of activated T cells, thereby compromising the antitumor immunity and reducing the effectiveness of immune-based therapies.^5^

Atypical Chemokine Receptor 2 (ACKR2), formerly known as D6, is a non-signaling chemokine receptor that regulates immune responses by scavenging and degrading inflammatory chemokines.^6^ Unlike classical chemokine receptors, which mediate intracellular signaling and direct immune cell migration, ACKR2 is unable to trigger canonical G protein-coupled signaling. Instead, it functions as a scavenger receptor, binding pro-inflammatory chemokines such as CCL2, CCL4, CCL5, CCL22, and CXCL10 facilitating their uptake and degradation, and thereby shaping the chemokine landscape within tissues.^7^ Predominantly expressed on lymphatic endothelial cells, trophoblasts, and certain immune cell subsets, ACKR2 plays a key role in limiting excessive inflammation and maintaining immune homeostasis by preventing chemokine accumulation.^8^ However, in cancer, ACKR2 has been implicated in tumor progression and immune evasion by modulating immune cell infiltration.^9^ Recent studies highlight the crucial role of atypical chemokine receptors (ACKRs) in controlling chemokine availability through their scavenging activity, positioning them as promising targets for immunotherapy.^10–12^ Chemokines associated with T cell-inflamed signatures, including CCL2, CCL4, CCL5, and CXCL10, are all scavenged by ACKR2, which is expressed by both tumor and endothelial cells.^7^ Based on this, we hypothesize that targeting *Ackr2* could help induce an inflamed tumor microenvironment, enhance cytotoxic immune cell recruitment, and improve the efficacy of anti-PD-1 immunotherapy.

## Materials and methods

### Cells and reagents

B16-F10 melanoma cells were purchased from ATCC and cultured following the guidelines provided by ATCC and a previous report.^13^ Cells were maintained at 37°C with 5% CO₂ in an incubator and regularly tested for mycoplasma contamination using the MycoAlert kit (Lonza). Control and *Ackr2* siRNAs were obtained from ThermoFisher, and control and *Ackr2* shRNAs were purchased from Santa Cruz Biotechnology. Mouse CCL5/RANTES and IFN-γ DuoSet ELISA kits were acquired from R&D Systems. The RT^2^ qPCR Primer Assay for mouse *Ackr2* was purchased from Qiagen. InVivoMab anti-mouse PD-1 (CD279) antibody (BE0273) and InVivoMab rat IgG2a isotype control (BE0089) were obtained from BioXCell (Lebanon, NH, USA). PE-conjugated anti-ACKR2 antibody was purchased from LSBio. Goat polyclonal anti-chemokine mouse receptor D6 (ACKR2) antibody (ab1656) and goat polyclonal IgG isotype control were obtained from Abcam. Rabbit anti-murine RANTES (CCL5) antibody (500-P118) and normal rabbit IgG isotype control were purchased from PeproTech.

### RNA extraction and SYBR green real-time (RT)-qPCR

Total RNA was extracted using TRIzol reagent (Invitrogen) following the manufacturer’s instructions. 1 μg of total RNA was treated with DNase I and reverse transcribed into cDNA using the TaqMan Reverse Transcription Reagent (Applied Biosystems). mRNA expression levels were quantified using SYBR Green-based qPCR (Applied Biosystems). Relative gene expression was determined using the comparative Ct method (2^−ΔΔCt^). Primer sequences are available upon request.

### Tumor immune phenotyping and flow cytometry analysis

Tumors were harvested, mechanically dissociated into fragments (<4 mm), and enzymatically digested using a mouse tumor dissociation kit (Miltenyi Biotec) for 45 minutes at 37°C. Single-cell suspensions were prepared, and red blood cells were lysed using ACK buffer (10-548E, Lonza). The cells were then counted with a Countess Automated Cell Counter (Invitrogen) and incubated on ice for 30 minutes with Fc block (TruStain fcX™ anti-mouse CD16/32 antibody 101,320, BioLegend). Samples were stained for surface markers associated with lymphoid immune populations, followed by intracellular staining for FoxP3 using the True-Nuclear™ Transcription Factor Buffer Set 424,401 (BioLegend) according to the manufacturer’s protocol.

The following antibodies were obtained from BioLegend: FITC anti-mouse CD45, Brilliant Violet 785 anti-mouse CD3, APC anti-mouse CD8a, APC/Fire 750 anti-mouse CD4, PE/Cy7 anti-mouse NK-1.1, Brilliant Violet 605 anti-mouse CD69, PE/Cy5 anti-mouse CD25, and Brilliant Violet 421 anti-mouse FOXP3. Viability staining was performed using a LIVE/DEAD Fixable Blue Dead Cell Stain Kit (ThermoFisher Scientific). Compensation controls were established using single dye stains, and fluorescence spread was assessed with FMO controls. Non-specific binding was evaluated using isotype controls. The gating strategies employed for immune phenotyping are detailed in.^13^

### In vivo study approval

Animal experiments were conducted in accordance with European Union guidelines. The in vivo experimental protocols received approval from the LIH Ethical Committee, the Animal Welfare Society, and the Luxembourg Ministry of Agriculture, Viticulture, and Rural Development (agreement no. LECR-2018-12).

### In vivo tumor growth and mouse treatments

C57BL/6 mice (7 weeks old) were purchased from Janvier-Labs and housed in pathogen-free conditions for one week prior to experimentation. The mice were injected subcutaneously into the right flank with B16-F10 cells (0.2 × 10^6^ cells) suspended in 100 µl of PBS. Anti-mouse PD-1 (CD279) (BE0273) and rat IgG2a isotype control (BE0089) were diluted in InVivoPure pH 7.0 Dilution Buffer (IP0070) and administered at 1 mg/kg as described in.^13^ On day 9, once tumors became palpable, mice were randomized into groups with equivalent average tumor sizes. Treatment with either anti-ACKR2 antibody, anti-CCL5 antibody, or a rabbit immunoglobulin isotype control was administered via intraperitoneal (IP) injection, beginning on day 10. Anti-CCL5 was administered at a dose of 2.5 mg/kg in 100 µl PBS, while 75 µg of anti-ACKR2 was given every other day until day 17 or 19. Tumor volume was measured every other day using calipers and estimated using the formula: Volume (cm^3^ = (width)^2^  × length × 0.5. Mice were excluded from the study if they failed to develop tumors or if the tumors exceeded the threshold defined in the approved experimental protocols (volume >2000 mm^3^), as previously reported.^13^

### Melanoma patient data mining

Data from the TCGA skin cutaneous melanoma (SKCM) cohort, comprising 448 patients, were downloaded from cBioPortal (http://www.cbioportal.org/). IDs of all patients were extracted, including those with BRAF and NRAS mutations, based on high, low and unaltered mRNA expression levels of *ACKR2*, *CCL5*, and *CXCL10* (z-scores relative to all samples). Each patient’s vital status and survival data (overall survival, and disease-specific survival) were obtained from the TCGA database. For patients categorized by high and low *CCL5/CXCL10* expression, the log2 mRNA expression levels (batch normalized from Illumina HiSeq_RNASeqV2) of CD8 markers (*CD8A* and *CD8B*) were identified. Differential expression of the genes of interest was analyzed using GraphPad software. The median survival and corresponding p-values were determined using the log-rank (Mantel-Cox) test in GraphPad. Cox proportional hazards regression analysis was conducted using GraphPad software to assess the impact of high versus low *ACKR2* expression, as well as high versus low *CCL5* and *CXCL10* levels, on survival outcomes. Expression data for *CCL5* and *CXCL10* in CTLA-4 and anti-PD-1 responders and non-responders among melanoma patients were obtained from.^14^

### Statistical analysis

Statistical analyses were performed using GraphPad Prism 10. An unpaired two-tailed t-test (parametric) was used to determine p-values between indicated groups. Results are represented as the mean ± standard error of the mean (SEM). A p-value <0.05 was considered statistically significant (*p* ≤ 0.05 = *; *p* ≤ 0.01 or ≤ 0.05 = **; *p* ≤ 0.001 or ≤ 0.005 = ***; *p* > 0.05 = not significant, ns).

## Results

### Genetic targeting of *Ackr2* in melanoma cells enhances chemokine release and suppresses tumor growth in immunocompetent mice

We first investigated the effects of genetically targeting *Ackr2* on the release by tumor cells of two chemokines, CCL5 and CXCL10, which can be scavenged by ACKR2^7^ and have high affinity for this receptor.^15^

We previously demonstrated that B16-F10 melanoma cells, which are resistant to anti-PD-1 therapy in vivo, exhibit relatively low expression of CCL5 and CXCL10, two key chemokines essential for recruiting NK and CD8^+^ T cells to the tumor microenvironment. However, the expression CCL5 and CXCL10 can be enhanced through various strategies^13,16,17^

**Figure 1.**
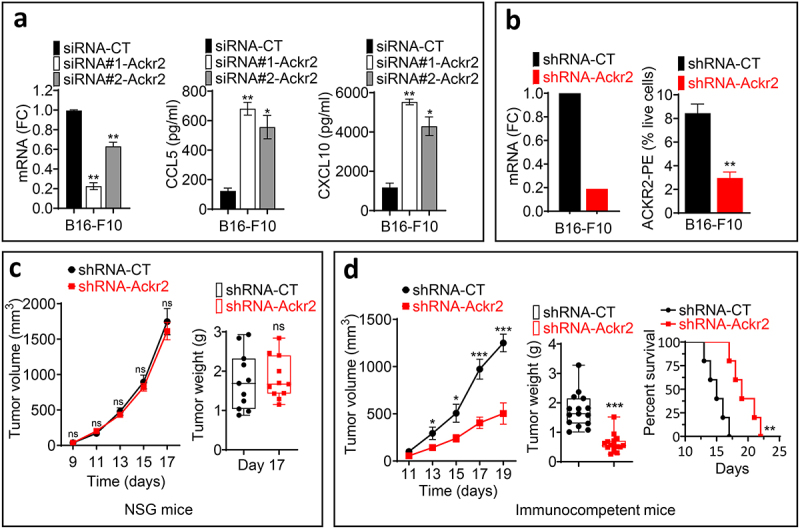
(a) mRNA expression levels of *Ackr2* [expressed as Fold change (FC) relative to control, left panel] and the secretion of CCL5 and CXCL10 [measured by ELISA, reported in pg/ml, middle and right panel respectively] in mouse melanoma B16-F10 cells transfected with control siRNA (siRNA-CT) or two distinct *Ackr2*-specific siRNAs [siRNA#1-Ackr2 and siRNA#2-Ackr2]. Only statistically significant differences are indicated. *p* < 0.05 (*); *p* < 0.01 (**). (b) mRNA expression of *Ackr2* [reported as Fold change (FC) relative to control, left panel] and its cell surface expression [reported as the percentage of live cells, right panel] in B16-F10 cells transfected with control shRNA (shRNA-CT) or *Ackr2* shRNA (shRNA-Ackr2). Only statistically significant differences are indicated. *p* < 0.01 (**) (c) tumor growth curves (left panel) and tumor weight [in grams (g)] on day 17 (right panel) of B16-F10 melanoma cells transfected with shRNA-CT or shRNA-Ackr2 and transplanted into NSG mice. Results are reported as the average of 11 mice per group from two independent experiments, one conducted with five mice per group and the other with six mice per group. Statistically significant differences are calculated using an unpaired two-tailed Student’s t-test (parametric). Data are presented as mean ± SEM (error bars). *p* ≥ 0.05 (not significant, ns) (d) tumor growth curves (left panel), tumor weight [in grams (g)] on day 19 (middle panel), and survival rates (right panel) of C57BL/6 immunocompetent mice bearing B16-F10 melanoma cells transfected with shRNA-CT or shRNA-Ackr2. Results are reported as the average of 15 mice per group from three independent experiments each conducted with five mice per group. Statistically significant differences are calculated using an unpaired two-tailed Student’s t-test (parametric). Data are presented as mean ± SEM (error bars). Only statistically significant differences are indicated. *p* < 0.05 (*); *p* < 0.001 (***). Survival curves (right panel, based on 5 mice per group) were generated from tumor-bearing mice, where lack of survival was defined as either death or a tumor size exceeding 1000 mm^3^. Survival percentages were calculated using GraphPad Prism, and p-values were determined using the log-rank (mantel-cox) test (** = p ≤ 0.01).

Using two different siRNA sequences targeting *Ackr2*, we observed a significant increase in the release of CCL5 and CXCL10 in *Ackr2*-knockdown cells compared to controls (Figure 1(a)).

We next assessed tumor growth in both control and *Ackr2*-targeted B16-F10 cells, generated using shRNA, in immunodeficient NOD SCID gamma (NSG) mice, which lack T, B, and NK cells, and in immunocompetent C57BL/6 mice. *Ackr2* knockdown in B16-F10 cells resulted in a significant reduction in both *Ackr2* mRNA expression and surface protein levels (Figure 1(b)). In NSG mice, Ackr2 targeting did not affect tumor growth or weight (Figure 1(c)). However, in immunocompetent mice, *Ackr2*-targeted tumors showed a marked reduction in growth and weight, which translated into significantly improved overall survival (Figure 1(d)).

### Targeting *Ackr2* enhances cytotoxic immune cell infiltration and inhibits tumor growth by modulating the release of key chemokines, primarily CCL5, in the tumor microenvironment

**Figure 2.**
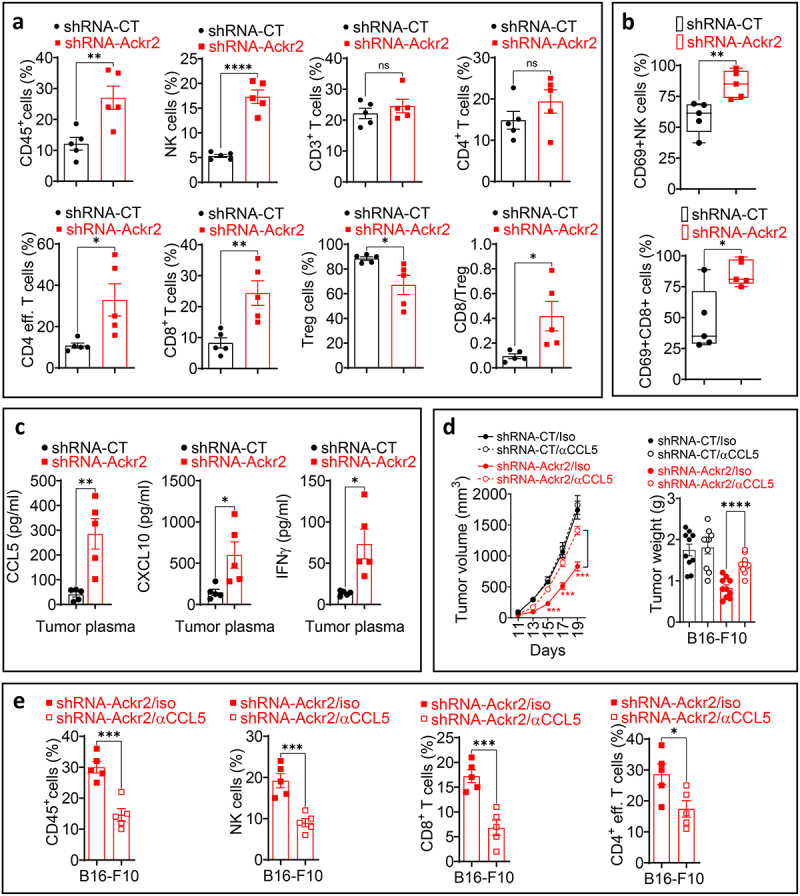
(a) flow cytometry analysis of CD45+ leukocytes (gated on live cells), NK cells, CD3+, CD4+, CD4+ effector (CD4 eff), CD8+ T cells, and Treg infiltrating shRNA-CT and shRNA-*Ackr2* B16-F10 tumors. Results are presented as the percentage of total cells. Treg (FoxP3+) infiltration was expressed as a percentage of CD4+ T cells in the tumor. The last panel shows the ratio of CD8+ T cells to tregs (CD8/Treg). Statistically significant differences are calculated using an unpaired two-tailed Student’s t-test (parametric). *p* ≥ 0.05 (not significant ns); *p* < 0.05 (*); *p* < 0.01 (**); *p* < 0.0001 (****). (b) quantification of the percentage of CD69+ activated CD8+ T cells and NK cells infiltrating tumors described in (A). Statistically significant differences are calculated using an unpaired two-tailed Student’s t-test (parametric). *p* < 0.05 (*); *p* < 0.01 (**). (c) ELISA quantification of CCL5, CXCL10, and IFN-γ released into the tumor microenvironment of shRNA-CT and shRNA-*Ackr2* B16-F10 tumors. Data are reported in pg/ml, normalized to excised tumor weight (g), and represented as the average of five tumors per group (each dot represents one tumor). Statistically significant differences are calculated using an unpaired two-tailed Student’s t-test (parametric). *p* < 0.05 (*); *p* < 0.01 (**). (d) tumor growth curves (left panel) and tumor weight in grams (g), (right panel), of shRNA-CT and shRNA-*Ackr2* B16-F10 tumors treated with either an isotype control antibody (Iso) or a CCL5-blocking antibody (αCCL5). Results are reported as the average of 10 mice per group from two independent experiments, each conducted with five mice per group. Statistically significant differences are calculated using an unpaired two-tailed Student’s t-test (parametric). Data are presented as mean ± SEM (error bars). Only statistically significant differences are indicated. *p* < 0.001 (***); *p* < 0.0001 (****). (e) flow cytometry quantification of CD45+, NK cells, CD8+ T cells, and CD4+ effector T cells infiltrating shRNA-*Ackr2* B16-F10 tumors treated as described in D. Statistically significant differences are calculated using an unpaired two-tailed Student’s t-test (parametric). *p* < 0.05 (*); *p* < 0.001 (***).

We next evaluated whether the tumor growth inhibition observed in *Ackr2*-targeted B16-F10 cells is associated with changes in immune cell infiltration, likely driven by altered availability of key chemokines such as CCL5 and CXCL10. Immune profiling of *Ackr2*-targeted B16-F10 tumors revealed a significant increase in the frequency of live CD45+ cells, CD4+ effector T cells, NK cells, and CD8+ T cells, along with a decrease in regulatory T cells (Tregs) compared to control (Figure 2(a)). Moreover, we observed an increased expression of the transient activation marker CD69 on NK and CD8+ T cells infiltrating *Ackr2*-targeted tumors (Figure 2(b)). However, assessing Granzyme B and IFN-γ levels in these cells would provide a more comprehensive evaluation of their activation state and cytotoxic potential. Nevertheless, these findings suggest that Ackr2 targeting enhances activated cytotoxic immune cell infiltration, likely by preventing the scavenging of key chemokines like CCL5 and CXCL10, which are essential for immune cell recruitment.^13,17^

This assumption is further supported by our data showing (i) increased levels of CCL5, CXCL10, and IFNγ in the tumor microenvironment of Ackr2-targeted tumors compared to controls (Figure 2(c)), and (ii) treatment with anti-CCL5-blocking antibodies in mice bearing *Ackr2*-targeted tumors, but not in those with control-targeted tumors, reverses the inhibition of tumor growth (Figure 2(d)) and decreases the frequency of CD45+, NK, CD8+, and CD4+ effector cells in the TME (Figure 2(e)).

### Targeting ACKR2 enhances the efficacy of anti-PD-1 therapy in B16-F10 melanoma model

**Figure 3.**
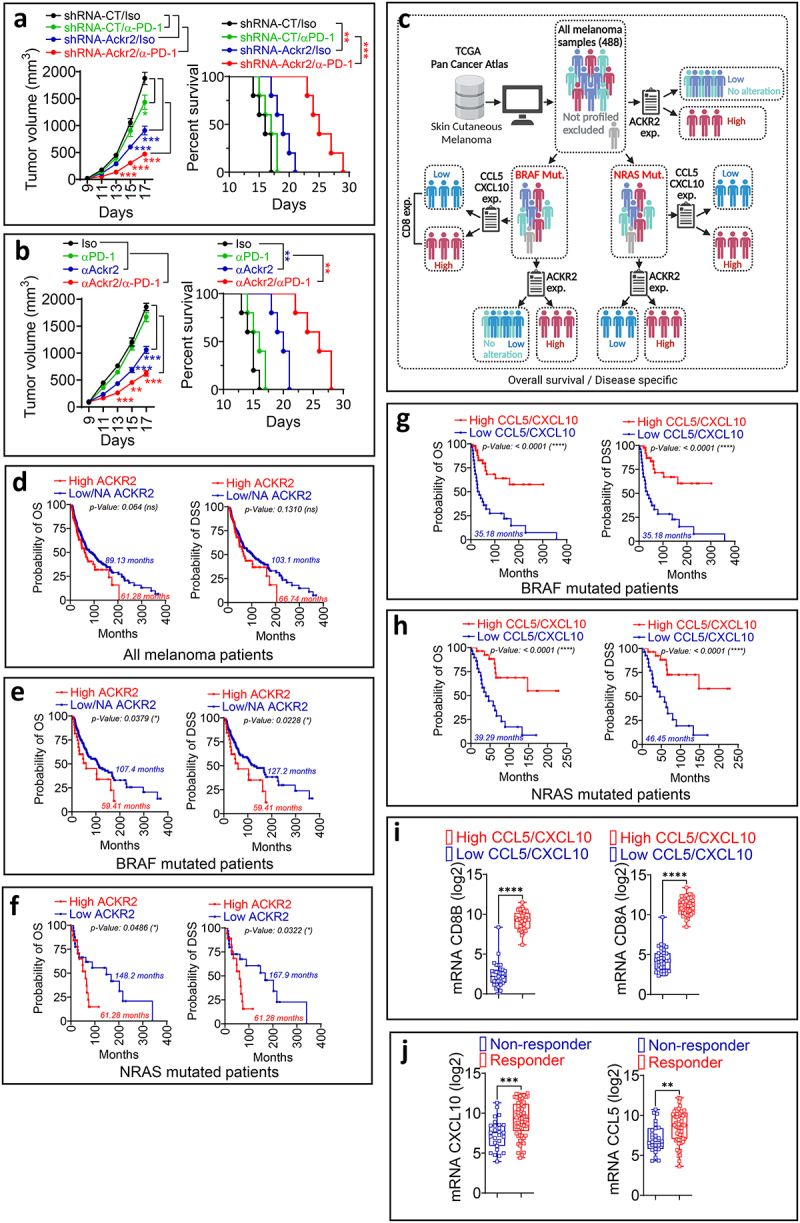
(a) tumor growth curves (left panel) and survival rates (right panel) of shRNA-CT and shRNA-*Ackr2* B16-F10 tumor-bearing mice treated with either an isotype control antibody (Iso) or anti-PD-1 antibody (α-PD-1). (b) tumor growth curves (left panel) and survival rates (right panel) of B16-F10 tumor-bearing mice treated with either isotype control (Iso), anti-PD-1 alone (α-PD-1), anti-ACKR2 blocking antibody (αACKR2) alone, or a combination of anti-ACKR2 and anti-PD-1 (αPD-1). For both panels (A and B), survival curves (5 mice per group) were generated from tumor-bearing mice treated as described. Lack of survival was defined as either death or tumor size exceeding 1000 mm^3^. Survival percentages were calculated using GraphPad Prism, with P-values determined by the log-rank (mantel-cox) test (ns = not significant; ** = p ≤ 0.01; *** = p ≤ 0.001). For panels A and B, results are reported as the average of 10 mice per group from two independent experiments, each conducted with five mice per group. Data are presented as mean ± SEM (error bars). Statistically significant differences in tumor growth were determined using an unpaired two-tailed Student’s t-test (parametric). Only statistically significant differences are indicated (** = p < 0.005; *** = p < 0.0005). (c) workflow established for analyzing melanoma patient data from the TCGA database, as detailed in the materials and methods section. (d, e and f) Kaplan-Meier survival curves depicting overall survival (OS, left panel) and disease-specific survival (DSS, right panel) in melanoma patients. Panel D includes all melanoma patients, while panel E focuses on those with BRAF mutations, and panel F on those with NRAS mutations. Patients are stratified based on *ACKR2* mRNA expression levels: high vs. low/not altered (NA) in panels D and E, and high vs. low in panel F. (g and h) Kaplan-Meier survival curves depicting overall survival (OS, left panel) and disease-specific survival (DSS, right panel) for melanoma patients with BRAF (G) and NRAS (H) mutations, classified by high and low mRNA expression levels of CCL5/CXCL10. The p-value for each Kaplan-Meier curve presented in panels D to H was calculated using the log-rank (mantel-cox) test. Cox proportional hazards regression values for ACKR2 (panel E) and CXCL5/CXCL10 (panel G) Kaplan-Meier survival curves in BRAF-mutated melanoma patients are presented in supplementary table S1. (I) the mRNA expression levels of CD8 markers (CD8A and CD8B) are shown as log2 values in melanoma patients with either low or high expression of CCL5 and CXCL10. Data are presented as mean ± SEM (with error bars). (j) the mRNA expression levels of CXCL10 and CCL5, presented as log2 values, in melanoma patients who responded to anti-PD-1 monotherapy or a combination of anti-PD-1/anti-CTLA4 therapy, as reported in.^14^ for panel I and J, statistically significant differences are calculated using an unpaired two-tailed Student’s t-test (parametric). *p* < 0.01 (**); *p* < 0.001 (***); *p* < 0.0001 (****).

Based on these results, we next investigated the effects of genetic targeting and pharmacological inhibition of ACKR2 on the efficacy of anti-PD-1 therapy in the B16-F10 melanoma model. As anticipated, anti-PD-1 monotherapy had minimal to no impact on tumor growth or survival in this model (Figure 3(a,b)). However, both genetic targeting (Figure 3(a)) and pharmacological inhibition of ACKR2 with an anti-ACKR2 antibody (Figure 3(b)) significantly reduced B16-F10 tumor growth. Interestingly, the therapeutic benefit of anti-PD-1 was markedly enhanced in mice bearing *ACKR2*-targeted B16-F10 tumors and in those treated with an anti-ACKR2 blocking antibody, resulting in significantly improved survival outcomes (Figure 3(a,b)).

To further investigate the role of ACKR2 in human melanoma, we analyzed its impact on patient survival using data from The Cancer Genome Atlas (TCGA). The melanoma patient data pipeline was retrieved and processed as outlined in Figure 3(c). While the difference observed in overall survival (OS) or disease-specific survival (DSS) was not significant between patients with high versus low *ACKR2* expression across all melanoma cases (Figure 3(d)), the prognostic relevance of *ACKR2* became evident in BRAF- and NRAS mutated melanoma (Figure 3(e,f)). Indeed, in BRAF and NRAS mutated subgroups, patients with low *ACKR2* expression or high levels of *CCL5* and *CXCL10* had significantly longer survival compared to those with high *ACKR2* expression or low levels of these chemokines (Figure 3(g,h)). Cox Proportional Hazards Regression values for *ACKR2* and *CCL5/CXCL10* for BRAF mutated melanoma patients are provided in Supplementary Table S1. This improved survival may be associated with an increased frequency of CD8+ T cells, as patients with elevated *CCL5* and *CXCL10* levels showed higher expression of the CD8 markers *CD8A* and *CD8B* (Figure 3(i)). Melanoma patient data corresponding to Figure 3(d-i) are available in Supplementary Tables S2 to S17.

These findings suggest that elevated levels of CCL5 and CXCL10, which are scavenged by ACKR2, may predict better outcomes in melanoma and an enhanced response to ICB-based therapy. This assumption is supported by data showing that melanoma patients who responded to anti-PD-1 therapy had significantly higher expression of both chemokines (Figure 3(j)), underscoring their potential role in driving a favorable therapeutic response. Melanoma patient data corresponding to Figure 3(h) are available in Supplementary Table S18.

## Discussion

In melanoma, T cell infiltration is strongly associated with a distinct chemokine gene signature that includes CCL2, CCL3, CCL4, CCL5, CXCL9 and CXCL10.^5^ This signature underscores the complexity of the chemokine network, which collectively shapes the immune landscape within the tumor microenvironment (TME) by promoting lymphocyte infiltration. Proper release of these chemokines is crucial for recruiting activated T cells, and any dysregulation can impair this process, thereby impacting the anti-tumor immune responses. Therapeutic strategies aimed at modulating the release of these key chemokines hold significant promise in converting “cold” tumors, characterized by low immune cell infiltration, into “hot” tumors, which are more inflamed and responsive to immunotherapy.

Several cell types within the TME, including both immune and cancer cells, are significant producers of the chemokines CCL5 and CXCL10. In esophageal squamous cell carcinoma, high levels of CCL5 and CXCL10 expression have been reported.^18^ In melanoma, up to 69% of cancer cells expressing the Sox10 transcription factor also express CXCL10,^19^ with similar expression patterns observed in hepatocellular carcinoma (HCC).^20^ In melanoma, CCL5 and CXCL10 play a critical role in recruiting CD8+ T cells and modulating responses to ICB therapies, including anti-PD-1 and anti-CTLA-4.^21^ Our findings further align with published data, demonstrating that high expression levels of CCL5 and CXCL10 are strongly associated with favorable patient outcomes and serve as valuable predictive markers for immunotherapy responses.^19^

*ACKR2* is expressed by a range of cell types, including hematopoietic precursor cells, lymphatic endothelial cells, and specific leukocyte populations such as innate B cells and alveolar macrophages.^9^ In addition, cancer-associated fibroblasts (CAFs), particularly in breast cancer, express ACKR2.^22^ While *ACKR2* expression in cancer cells is generally low, it can still be detected in certain cancers, such as anaplastic thyroid carcinoma.

Given that multiple cell types within the TME produce CCL5 and CXCL10 and express ACKR2, targeting ACKR2 could have a substantial impact. As a result, this strategy could shift the immune landscape toward a more pro-inflammatory and immune-active state, ultimately enhancing the effectiveness of immunotherapies. In this study, we present experimental evidence supporting the concept of ACKR2 as a promising therapeutic target. By enhancing the levels of chemokines that attract CD8+ T cells, targeting ACKR2 may improve the efficacy of anti-PD-1 therapy.

Although ACKR2 was initially reported to scavenge only C-C chemokines such as CCL5, we have demonstrated that it can also scavenge CXC chemokines, including CXCL10.^15,23^ Thus, unlike ACKR5/GPR182,^6,24^ which scavenges CXCR3-related chemokines^25^ and not CCL5, ACKR2 represents a promising target for simultaneously modulating the bioavailability of both CCL5 and CXCL10 however, the affinity of ACKR2 for different chemokines varies, with ACKR2 displaying a particularly high affinity for CCL5. As a result, ACKR2 preferentially scavenges CCL5, which is produced by various cells within the TME. The data presented in this manuscript focus on CCL5 and CXCL10 as key indicators for evaluating the impact of regulating the expression of ACKR2 in melanoma model. The rationale for this approach is based on the fact that the B16-F10 model used in this study predominantly releases CCL5 and CXCL10. However, we believe that regulating ACKR2 expression may also influence other chemokines that are known to be scavenged by ACKR2 and play a role in attracting critical cytotoxic immune cells to the TME such as CCL2, CCL4, CCL7, and CCL8. Given the pivotal role of ACKR2 in regulating the bioavailability of inflammatory chemokines within the TME, understanding the mechanisms that control its expression is also crucial. Factors in the TME, such as hypoxia, can induce *ACKR2* expression in different cells. Our recent data demonstrate that hypoxia-inducible factor-1 alpha (HIF-1α) directly binds to the *Ackr2* promoter, which contains multiple hypoxia-responsive elements (HREs), thereby inducing its expression under hypoxic conditions.^26^ This increase in ACKR2 could, in turn, deplete pro-inflammatory chemokines and impact immune cell infiltration. Although further experimental validation is required to confirm this hypothesis, it may at least partially explain the immunosuppressive nature of hypoxic tumors.

The data presented here emphasize the potential of combining ACKR2 inhibition with ICB as a promising therapeutic strategy for melanoma. In particular, melanoma patients harboring BRAF and NRAS mutations may benefit from therapies targeting ACKR2. This is supported by our findings that low *ACKR2* expression correlates with improved survival in these patient subgroups. However, the relationship between ACKR2 expression and BRAF/NRAS mutations in melanoma remains unclear and warrants further investigation. Nevertheless, indirect connections can be proposed through mechanisms involving hypoxia and immune modulation. Notably, we recently reported that hypoxia, via HIF-1α stabilization, can directly upregulate *Ackr2*.^26^ Therefore, it is essential to determine whether BRAF- and NRAS-mutant melanomas exhibit increased hypoxia, which may explain the elevated *ACKR2* expression. If confirmed, targeting ACKR2 could represent a beneficial therapeutic approach in BRAF/NRAS-mutated melanoma patients.

Expanding research to investigate the impact of ACKR2 targeting in additional tumor models would provide broader insights into its therapeutic applicability. Remarkably, targeting ACKR2 with small molecules or neutralizing antibodies could open new opportunities for treating “cold” tumors, which are typically resistant to immune checkpoint inhibitors. However, further investigations are required to determine the optimal integration of this approach with existing immunotherapies, enabling treatments to be personalized and based on the unique immune profile of each tumor.

Nevertheless, it is important to note that using ACKR2 inhibitors in clinical settings poses complex and context-dependent effects within the TME, as ACKR2 has been shown to play both tumor-suppressing and tumor-promoting roles.^27^ Its tumor-suppressive effect is primarily attributed to its ability to limit inflammation by scavenging pro-inflammatory chemokines, thereby reducing tumor-promoting chronic inflammation. This is particularly evident in models of colitis-associated cancer, where *Ackr2*-deficient mice exhibit more severe experimental colitis and an increased cancer incidence due to heightened inflammatory chemokine production and excessive leukocyte infiltration.^28^ In colon adenocarcinoma, reduced *Ackr2* expression correlates with elevated levels of CCL22, a chemokine that recruits regulatory T cells (Tregs), promoting an immunosuppressive tumor microenvironment. Notably, lower ACKR2 expression in colon adenocarcinoma tissues is linked to higher tumor grades (T3 and T4),^29^ further suggesting a potential tumor-suppressive function. Moreover, since ACKR2 regulates lymphatic vessel density and immune cell trafficking, its inhibition may lead to immune dysregulation and an increased risk of autoimmune reactions.^8,30^ Conversely, ACKR2 can also promote tumor progression by suppressing anti-tumor immune responses. By scavenging chemokines such as CCL2, it impairs the recruitment of key immune effectors like NK cells, thereby reducing tumor-killing activity and facilitating immune evasion.^31^ The impact of regulating ACKR2 varies depending on cancer type, stage, and the specific immune cells involved in the anti-tumor response. These contrasting roles underscore the complexity of chemokine regulation within the TME and suggest that while ACKR2 could serve as a potential therapeutic target, its modulation must be carefully tailored to the specific cancer context.^11,12^

We believe that achieving therapeutic efficacy with ACKR2 inhibition, while minimizing side effects, requires integrative approaches and a comprehensive understanding of the unique immune context and chemokine profile of each tumor. This balanced approach is essential to harnessing ACKR2 as a valuable target in next-generation cancer immunotherapies.

## Supplementary Material

Supp table 2_.xlsx

Supp table 5.xlsx

Supp table 15.xlsx

Supp table 12.xlsx

Supp table 10.xlsx

Supp table 8.xlsx

Supp table 17.xlsx

Supp table 14.xlsx

Supp table 9.xlsx

Supp table 11.xlsx

Supp table 4.xlsx

Supp table 13.xlsx

Supp table 6.xlsx

Supp table 1.xlsx

Supp table 7.xlsx

Supp table 18.xlsx

Supp table 16.xlsx

Supp table 3_.xlsx

## Data Availability

The datasets used to support the findings of this study are available from the corresponding authors upon reasonable request.
